# Forensische Bildgebung der scharfen Gewalt

**DOI:** 10.1007/s00117-024-01370-5

**Published:** 2024-09-25

**Authors:** Peter Hofer, Christiane Ferling

**Affiliations:** 1 Diagnostik- & Forschungsinstitut für Gerichtliche Medizin, Medizinische Universität Graz, Neue Stiftingtalstraße 6, 8010 Graz, Österreich; 2 Institut für Rechtsmedizin und Verkehrsmedizin Heidelberg, Universitätsklinikum Heidelberg, Heidelberg, Deutschland

**Keywords:** Luftembolie, Stichverletzung, 3‑D-Rekonstruktion, Postmortale Computertomografie, Stichkanal, Air embolism, Stab wound, Three-dimensional reconstruction, Post-mortem computed tomography, Stab canal

## Abstract

**Problemstellung:**

Die Beantwortung juristischer Fragen ist in der Rechtsmedizin alltägliche Routine. Die Rekonstruktion von Gewaltdelikten nach Angriffen mit scharfen und/oder spitzen Werkzeugen ist zumeist komplex und nur mit klassischen rechtsmedizinischen Methoden, wie z. B. der Obduktion, kaum mehr adäquat zu beantworten. Aus diesem Grund ist die klinische und postmortale Radiologie zur idealen Ergänzung in der Rechtsmedizin geworden.

**Methoden und Verfahren:**

Während die klassische Röntgenuntersuchung für die Darstellung von Weichteilverletzungen ungeeignet ist und die Magnetresonanztomographie (MRT) zu teuer, zu aufwendig und auch nicht überall verfügbar ist, hat sich mittlerweile die Multislice-Computertomographie (MSCT) als Methode der Wahl in der forensischen Bildgebung bei der Beurteilung von Verletzungsfolgen scharfer Gewalt ergeben.

**Vorteile:**

Die forensische Bildgebung, insbesondere die MSCT, bietet insbesondere nach Stichverletzungen eine unverzichtbare Ergänzung in der Rekonstruktion. So ist sie bei der Beurteilung von Stichkanälen, der Rekonstruktion von Tatabläufen oder der Beurteilung von (lebensgefährlichen) Verletzungen in vielen Fällen der klassischen Obduktion zumindest gleichgestellt, teilweise auch überlegen.

**Empfehlung:**

Die forensisch-radiologische Begutachtung nach scharfen Gewalteinwirkungen erfordert Erfahrung sowohl auf dem Gebiet der Radiologie als auch der Rechtsmedizin. Eine enge Zusammenarbeit beider Disziplinen vorausgesetzt, kann sie ein wesentliches Mittel in der Versorgung von Gewaltopfern sein.

## Abstract (en)

**Background:**

Answering legal questions is everyday routine in forensic medicine. The reconstruction of violent crimes following attacks with sharp and/or pointed instruments is usually complex and can hardly be adequately answered using traditional forensic methods such as autopsies. For this reason, clinical and postmortem radiology has become the ideal complement to forensic medicine.

**Methods:**

While classic X‑ray is unsuitable to image soft tissue injuries, and magnetic resonance imaging is too expensive, too complex and also not available everywhere, multislice computed tomography (MSCT) has now emerged as the method of choice in forensic imaging for the assessment of the consequences of sharp force injuries.

**Achievements:**

Forensic imaging, especially MSCT, offers an indispensable addition to reconstruction, particularly after stab wounds. In many cases, it is at least equal to, and in some cases superior to, the classic autopsy in the assessment of stab wounds, the reconstruction of the sequence of events or the assessment of (life-threatening) injuries.

**Conclusions:**

The forensic radiological assessment after sharp violence requires experience in both the field of radiology and forensic medicine. Provided that both disciplines work closely together, forensic radiology can be an essential tool in the care for victims of violence.

Die Beurteilung scharfer Gewalteinwirkungen gegen den menschlichen Körper gehört zur täglichen Routine in der Rechtsmedizin. Bildgebende Verfahren haben sich hierzu mittlerweile als unverzichtbar erwiesen, da sie die Beantwortung vieler rechtlich relevanter Fragestellungen nicht nur erleichtern, sondern diese oftmals überhaupt erst ermöglichen – insbesondere zur Rekonstruktion von Stichverläufen und resultierenden Verletzungen. Da hier jedoch eben nicht die klinische Versorgung im Vordergrund steht, ist eine entsprechende forensische Expertise auf diesem Gebiet für Radiolog*innen unerlässlich.

## Definitionen

Zur Verletzungsentstehung durch scharfe Gewalt bedarf es der Einwirkung von spitzen und/oder scharfen Werkzeugen, wie etwa durch Messer, Scheren, Nadeln, Schraubenzieher, Glassplitter/-scherben etc.

Zu unterscheiden sind zunächst Schnitt- und Stichverletzungen. Bei Letzteren handelt es sich um Gewebsdurchtrennungen durch zumeist spitz zulaufende Werkzeuge, welche unter Ausbildung eines Stichkanals überwiegend senkrecht und/oder schräg zur Körperoberfläche geführt werden. Schnittverletzungen dagegen werden demgegenüber durch überwiegend parallel und/oder tangential zur Körperoberfläche geführte, scharfe Werkzeuge hervorgerufen [6]. Ihnen gemeinsam sind morphologische Kriterien der scharfen Gewalteinwirkung wie glatte, zumeist gerade Wundränder, das Fehlen von Gewebebrücken in der Tiefe sowie zumeist eines Schürf- oder Vertrocknungssaums. Besonders bei mit hoher Wucht ausgeführten Stichen kann es im Randbereich der Hautdurchtrennung in Einzelfällen auch zu Quetschungen durch Einstich bis zum Messerheft kommen [6]. Einen Sonderfall bildet hier die sog. halbscharfe Gewalt, welche klassischerweise Hiebverletzungen hervorruft. Typische Tatwerkzeuge sind hierbei Äxte und Beile, Säbel, Macheten, aber auch Propeller oder Schiffsschrauben. Neben dem schneidenden Aspekt des Tatwerkzeugs, spielt auch die hohe Wucht bzw. das große Eigengewicht eine erhebliche Rolle in der Verletzungsentstehung. Dadurch kommt es regelmäßig neben der klassischen, einer scharfen Gewalteinwirkung zuordenbaren Wundmorphologie auch zu Merkmalen der stumpfen Gewalt, wie etwa Quetschungen und Schürfungen. Die Wundränder sind auch hier in der Regel glatt, und es fehlen Gewebsbrücken in der Tiefe. Der Wundquerschnitt kann keilförmig imponieren [6, 14].

## Juristische und rechtsmedizinische Fragestellungen

Zur Beantwortung der zumeist sehr allgemein an die Rechtsmedizin gestellten Fragen der Ermittlungsbehörden im Rahmen des Ermittlungsverfahrens ist für die Rechtsmediziner*innen in der Folge eine Reihe weiterer, detaillierterer Fragen zu beantworten (Tab. 1).

**Tab. 1:** Fragen der Ermittlungsbehörden bzw. Fragen der Rechtsmedizin

Fragestellungen von Ermittlungsbehörden	Fragestellungen der Rechtsmedizin
Welche Verletzungen sind entstanden?	Anzahl und Lokalisation der Verletzungen?
Lebensgefahr? (Bei überlebten Fällen) Welche Verletzungen waren tödlich?	Wurden lebenswichtige Strukturen verletzt?
Welches Tatwerkzug wurde verwendet?	Lässt sich ein Stichkanal rekonstruieren?
	Lässt sich aus dem Verletzungsmuster die Klingenbeschaffenheit rekonstruieren?
Stichwucht	Durchstechen fester Strukturen, z. B. Schädel oder Brustbein?
	Fremdköper in der Wunde, z. B. abgebrochene Klinge?
Selbst- oder Fremdbeibringung?	Abwehrverletzungen?
Handlungsfähigkeit?	Vitalitätszeichen?
	Verletzungen die sofortige(n) Handlungsunfähigkeit bzw. Tod begründen?
Dauerfolgen?	Amputationen?
	Funktionseinschränkungen?
	Narben?
–	Verletzungen an Knochen oder Gelenken?
–	Alternative Todesursachen?
–	Sonstige Gewalteinwirkungen?

## Bedeutung der forensischen Bildgebung

Je nach Fallkonstellation können von juristischer Seite weitere Fragenkomplexe relevant werden. Postmortal lassen sich zwar durch sorgfältige Präparation im Rahmen der Obduktion viele dieser Fragen auch aufgrund der makroskopisch erhebbaren Befunde rekonstruieren und beantworten. Naturgemäß aber geht mit jeder Präparation eine Manipulation der ursprünglichen Wundverhältnisse einher, was die Gefahr einer Verfälschung und Fehlinterpretation von Befunden birgt. Daher stellt die postmortale Computertomographie (PMCT) ein mittlerweile nahezu unverzichtbares Instrument zur rechtsmedizinischen Diagnostik und Tatrekonstruktion dar, da sie den Status quo vor Beginn der Obduktion sichert.

Zudem lassen sich auch im Nachgang aufgetretene Fragen beantworten, die erst aufgrund z. B. ergänzender Informationen nach Zeug*innenbefragungen aufkommen. Neben der klassischen postmortalen Computertomographie (PMCT) gewinnen auch die postmortale Computertomographie-Angiographie (PMCTA) sowie 3‑D-Oberflächenscans zur späteren Verletzungsbegutachtung und Rekonstruktion zunehmend an Bedeutung.

Doch nicht nur postmortal, ergänzend zur klassischen Obduktion, sondern auch in der klinischen Rechtsmedizin am Lebenden ist die forensische Radiologie bedeutsam. Oftmals können die in Tab. 1 genannten Fragen allein deshalb nicht beantwortet werden, weil nach scharfen Gewalteinwirkungen die körperliche Untersuchung zu spät beauftragt wird oder überhaupt nicht stattfindet. In solch einem Fall ist eine Beurteilung nur noch anhand klinischer Angaben und insbesondere der präoperativen Bildgebung möglich. Grundsätzlich sind die postmortale Bildgebung und die klassische rechtsmedizinische Leichenschau und Obduktion bzw. körperliche Untersuchung nach überlebten Delikten als komplementäre Methoden zur Beurteilung scharfer Gewalteinwirkungen zu betrachten (Tab. 2).

**Tab. 2:** Typische Verletzungsbefunde (halb)scharfer Gewalteinwirkungen. (Nach [13])

Gewaltform	Makroskopische Befunde	Radiologische Befunde
Stich	Glatte, gerade Wundränder Fehlen von Gewebebrücken Einblutungen im Wundbereich Ausbildung eines Stichkanals Zumeist tiefer als breit	Defekt der Haut als Hautdurchtrennung/Kontinuitätsunterbrechung erkennbar Einblutungen und Lufteinschlüsse und ggf. Weichteildefekt in Unterhautfett‑, Weich- und Organgewebe Evtl. Gefäßverletzungen, Verletzungen des Knochens bzw. des Knorpels Evtl. Eröffnung von Körperhöhlen mit Nachweis von freier Flüssigkeit, Pneumothorax, Pneumoperitoneum etc.
Schnitt	Glatte, gerade Wundränder Fehlen von Gewebebrücken oft ritzerartige Ausläufer Einblutungen im Wundbereich	Defekt der Haut als Kontinuitätsunterbrechung erkennbar Evtl. lokale Einblutungen und Lufteinschlüsse Evtl. Verletzungen von Weichgewebe und Strukturen
Halbscharf	Zumeist glattrandige Wundränder Schürfungen/Quetschungen möglich Umgebende Einblutungen Zeichen stumpfer Gewalteinwirkung (z. B. Frakturen)	Defekt der Haut als Kontinuitätsunterbrechung erkennbar Einblutungen und Lufteinschlüsse im Weichgewebe, ggf. bis zu Quetschungen Verletzungen des Knorpels bzw. des Knochens, mögl. Knochenzertrümmerungen, Knochensplitter Amputationen

## Bedeutsame radiologische Befunde

### Oberflächliche Verletzungen

Äußerlich sichtbare Verletzungen sind radiologisch in der PMCT zumeist als Kontinuitätsunterbrechungen der Haut erkennbar. Hierbei gilt es allerdings zu beachten, dass Hautdurchtrennungen und -verletzungen nicht zwingend radiologisch erkennbar sein müssen. Die Sichtbarkeit hängt u. a. von der Größe und der Lokalisation der Verletzung ab. So können Verletzungen am Rücken durch die Untersuchung in der CT in Rückenlage durch Kompression von Weichgewebe maskiert werden. Untersuchungen zeigen zudem, dass insbesondere sehr oberflächlich gelegene Verletzungen oftmals nicht, oder nur sehr schwer radiologisch zu erkennen sind. Auch eine Differenzierung mehrerer, in engem räumlichen Bezug zueinanderstehender Stich- und Schnittverletzungen kann sich schwierig gestalten, und eine Beurteilung sollte hier nur mit großer Vorsicht erfolgen [24].

**Abb. 1:**
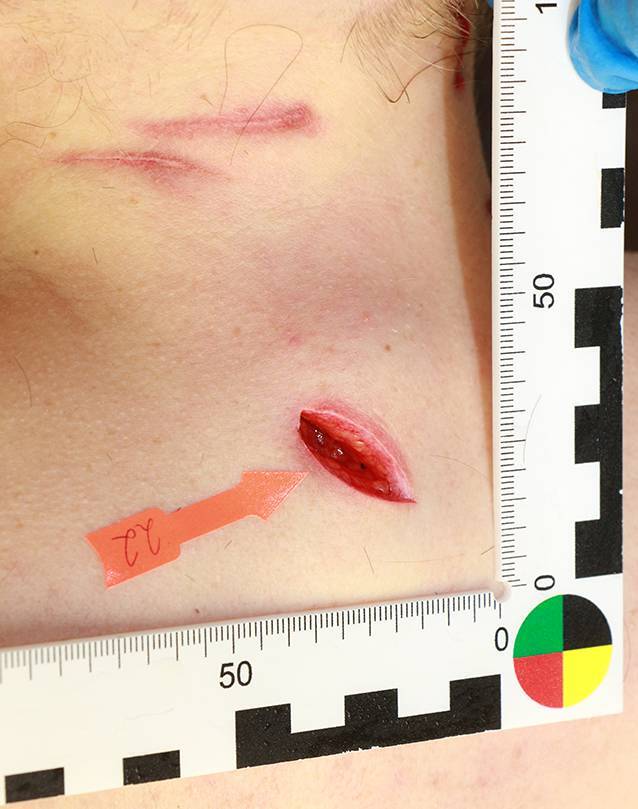
Stichverletzung Nackenregion (mit Nummer markiert, Ansicht von dorsal). Oberhalb 2 oberflächliche, ritzerartige Defekte nach scharfer Gewalt im Rahmen eines Tötungsdelikts mit insgesamt mehr als 20 Stich- und Schnittverletzungen

Oberflächliche Schnittverletzungen sind zumeist nur schwer radiologisch darstellbar. Die am besten geeignete Untersuchungsmethode stellt hier noch die Multislice-CT (MSCT) dar. Die klassische Röntgenaufnahme stellt durch ihre schlechte Darstellung von Weichteilgewebe keinen wesentlichen Erkenntnisgewinn dar. Magnetresonanztomographien (MRT) sind hierfür zu teuer und zu zeitaufwendig [9]. Zur späteren Rekonstruktion und Veranschaulichung, etwa im Rahmen einer Hauptverhandlung, kann von äußerlich sichtbaren Verletzungen ein 3‑D-Oberflächenscan erfolgen. Volumenrekonstruktionen (VRT) von MSCT erscheinen hierzu zwar grundsätzlich auch geeignet, erreichen jedoch derzeit noch nicht die Qualität alternativer Verfahren. Insbesondere die Beschreibung der Verletzungsform sowie der Wundwinkel und -ränder ist kaum möglich. Insofern sollte die Wundbeschreibung äußerlich sichtbarer Verletzungen im Rahmen der äußeren Leichenschau bzw. Obduktion erfolgen ([5, 13]; Abb. 1 und 2).

**Abb. 2:**
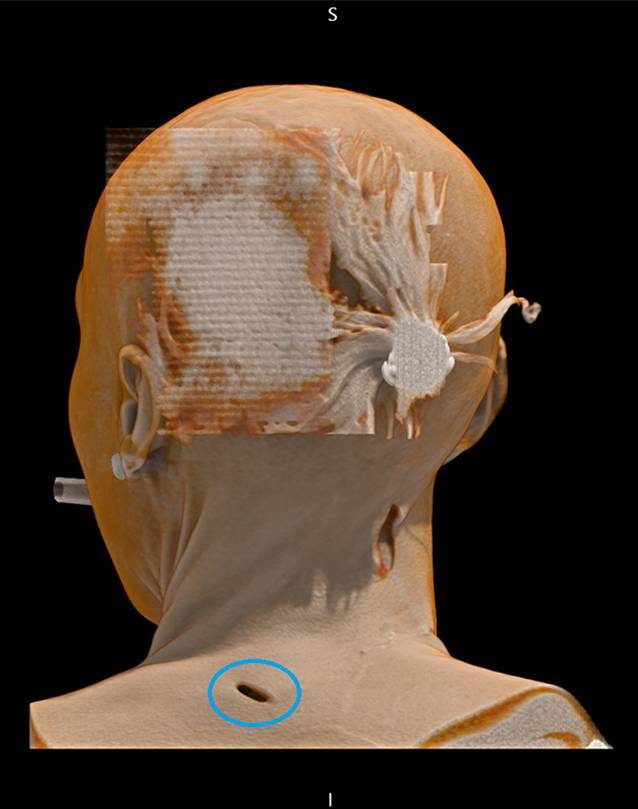
3‑D-Rekonstruktion der Stichverletzung aus Abb. 1 (*Kreis*)

### Stichverletzungen

Von wesentlich größerer Bedeutung sind die Befundung und Rekonstruktion innerer Verletzungen, wie sie im Regelfall bei Stichverletzungen auftreten. Hierbei hat sich die MSCT als Methode der Wahl erwiesen. Sie erlaubt eine gute Differenzierung zwischen unterschiedlichen Gewebetypen wie Haut, Organen, Knochen, Muskulatur oder Fettgewebe [9].

**Abb. 3:**
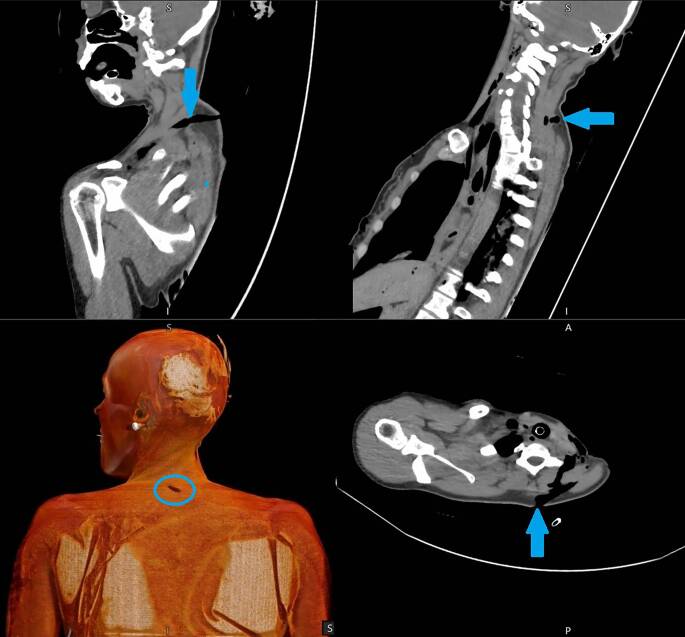
Postmortale Computertomographie (PMCT; selbe Person wie in Abb. 1) mit Darstellung des Stichverlaufs in allen Ebenen sowie 3‑D-Rekonstruktion des Hautdefekts (*Pfeile*). Ausbildung einer Lufttasche, jedoch ohne tiefere Verletzungen

Verletzungen von Gefäßen sind in der nativen (PM)CT schwer zu erkennen [23]. Hierbei ist die Obduktion der PMCT zumeist überlegen. Ein besserer radiologischer Nachweis kann in der aufwendigeren PMCTA erfolgen. Indirekte Hinweise liefern in der nativen Untersuchung angrenzende Gewebsveränderungen wie freie Flüssigkeit, Hämatome und Luftbläschen. Aus diesem Grund sollte bei Vorliegen von Stichverletzungen stets an die Möglichkeit einer CT-Angiographie gedacht werden, da diese eine erleichterte Detektion von Gefäß-, aber auch Organverletzungen erlaubt. Aufgrund des Austritts von Kontrastmittel, auch schon aus kleinen Gefäßen in das umgebende Gewebe, lässt sich hierbei oft auch der gesamte Stichkanal rekonstruieren [3, 18, 20, 23, 27]. Insbesondere in dreidimensionaler Rekonstruktion lassen sich Winkel, Richtung und Verlauf eines Stichkanals nachvollziehen. Hierdurch können auch die minimalen und maximalen Wundkanallängen bestimmt und somit mögliche Rückschlüsse auf das verwendete Tatwerkzeug gezogen werden, wie später noch ausführlich beschrieben. Hierzu ist eine Rekonstruktion in zumindest 2 der 3 Dimensionen (axial, koronar und sagittal) erforderlich ([9]; Abb. 3).

Unter bestimmten Umständen lässt sich anhand des Verletzungsbildes sogar die Abfolge der Stiche nachvollziehen.

Bei der Rekonstruktion von Wundkanälen müssen postmortale Veränderungen der Organposition durch Blutungen, Pneumothorax und Lungenkollaps berücksichtigt werden. Atem- und Herzbewegungen beeinflussen ebenfalls die Position der Organe zum Zeitpunkt des Traumas. Bewegungen der Beteiligten während des Angriffs sowie Weichteilemphyseme oder Blutansammlungen durch scharfes Trauma können die Messung der Wundtiefe verfälschen. Auch können bei Weichteilemphysemen diffuse Lufteinschlüsse eine solche Rekonstruktion erschweren oder verfälschen, da diese nicht immer in unmittelbarem räumlichem Bezug zu den Verletzungen liegen müssen. Die gemessene Wundkanallänge entspricht auch nicht zwangsläufig der Waffenlänge, da die Gewebeelastizität und -kompression die tatsächliche Eindringtiefe verfälschen können [4, 22]. Neben der Darstellung des Wundkanals kann eine ergänzende 3‑D-Rekonstruktion zur Veranschaulichung möglicher in der Nähe des Kanals gelegener lebenswichtiger Strukturen dienen, wie etwa Herz, Lunge, Aorta, Leber etc. Dies stellt in der späteren rechtlichen Wertung eine wichtige Beurteilungsgrundlage dar, insbesondere hinsichtlich der Gefährlichkeit einer Tathandlung.

### Organverletzungen

**Abb. 4:**
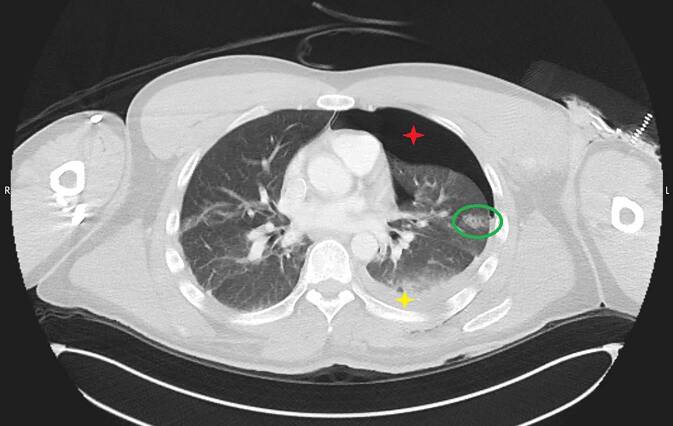
CT des Thorax, axial im Lungenfenster. Lungengewebsverletzung (*Kreis*) und ipsilateraler Hämatopneumothorax (*gelber* bzw. *roter Stern*) nach Auseinandersetzung mit einer Glasscherbe und Verletzung links thorakal (achselhöhlennah)

Die Beurteilung von Organverletzungen ist neben einer potenziellen Stichkanalrekonstruktion auch für die Beurteilung einer möglichen Lebensgefährdung von essenzieller Bedeutung. So führen Stichverletzungen gegen den Thorax regelmäßig zur Ausbildung eines Hämato- und/oder Pneumothorax. Kommt es zusätzlich zu einem Mediastinalshift und somit zum Spannungspneumothorax, ist im Regelfall von einer akut lebensbedrohlichen Situation auszugehen. Derartige Verletzungen sind sich in der Regel radiologisch leicht zu erkennen. Insbesondere wenn die Tat überlebt wurde, ist die radiologische Abschätzung des Blutvolumens im Thorax zur Einschätzung wichtig, ob tatsächlich Lebensgefahr bestand. Insofern ist es bedeutsam, Blut von möglichem Trans- oder Exsudat zu unterscheiden. Flüssiges Blut hat in der Computertomographie ca. 50 Hounsfield Units (HU), geronnenes Blut bis etwa 75 HU [12, 28]. Demgegenüber weisen Trans- bzw. Exsudat je nach möglichen Begleiterkrankungen Dichtewerte in einem Bereich von ca. 5 bis zu 33 HU auf ([8]; Abb. 4).

**Abb. 5:**
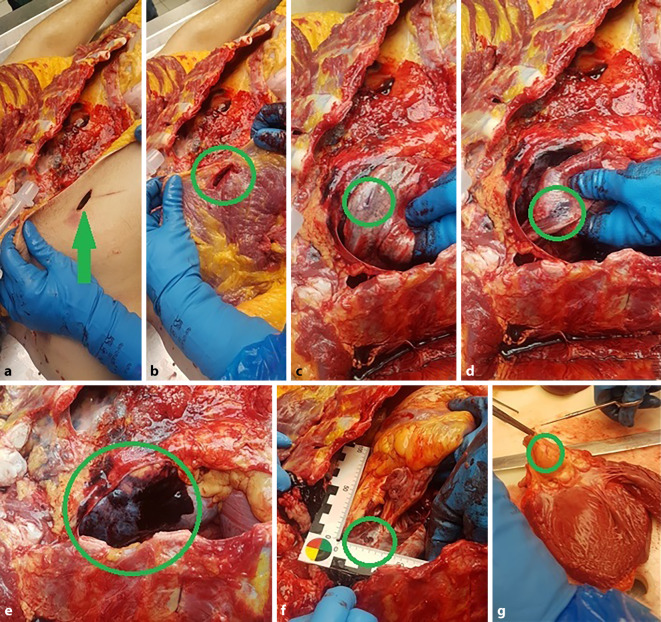
Obduktionsfotos mit Darstellung des Stichverlaufs (*Kreis*) mit Durchstich durch Haut (**a**), M. pectoralis (**b**), rechte Lunge (**c**–**e**), Perikard (**f**), Aorta (**g**)

Kommt es neben Lungen- auch zu Herzstichverletzungen, so ist neben der Blutung in die Brusthöhle auch an die Möglichkeit einer Herzbeuteltamponade zu denken. Hierbei können Volumina von ca. 250 ml bereits tödlich sein. Herzstichverletzungen können, abhängig von ihrer Lokalisation, auch längere Zeit noch überlebt werden [11, 17, 19, 25]. Neben den Blutmengen ist auch auf den Zustand der Herzkammern und großer Blutgefäße zu achten, da deren Kollaps darauf hinweisen, dass der Tod durch Verbluten eintrat [13]. Eine Ausnahme stellt hier etwa die Vena cava dar, wo ein derartiges Bild postmortal auch als Normalbefund auftreten kann (Abb. 5 und 6).

**Abb. 6:**
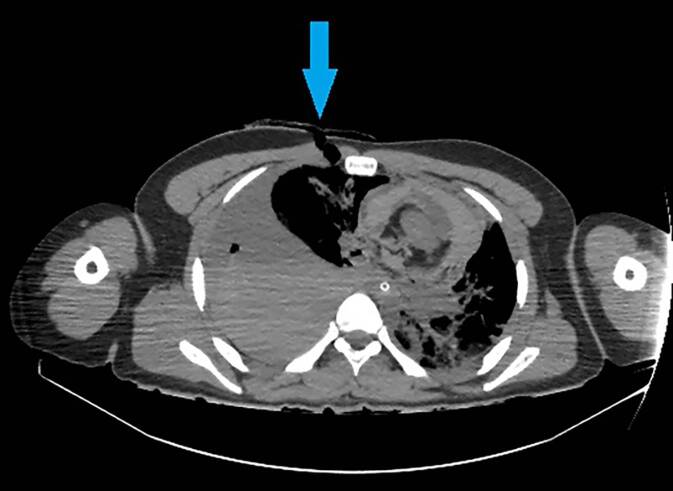
Zugehörige postmortale Computertomographie (PMCT) im Weichteilfenster, axial. Weichteildefekt an der rechten Brustkorbvorderseite (*Pfeil*). Ipsilateraler Hämatopneumothorax und Herzbeuteltamponade

Des Weiteren ist auch auf Gasansammlungen zu achten, da diese abhängig von Luftmenge und Lokalisation als Todesursache betrachtet werden können. Sie können auch belegen, dass zum Zeitpunkt der Verletzungsentstehung intakte Kreislaufverhältnisse bestanden, indem durch die Eröffnung von Venen – insbesondere im Halsbereich – durch den dort herrschenden Unterdruck Luft angesaugt wird. Luft ist in der CT mit ca. −1000 HU gut zu erkennen. Bereits eine Gasmenge zwischen 70 und 300 cm^3^ kann letal sein [1, 21, 26]. Intravasale Luftvolumina im niedrigen zweistelligen Bereich können hingegen vernachlässigt werden [15]. Insbesondere beim Nachweis der Luftembolie ist die PMCT der klassischen Obduktion überlegen [24]. Falls bereits Fäulnis eingetreten ist, sollte die Möglichkeit von Fäulnisgasen bedacht werden, die sich im Gegensatz zur Luftembolie zumeist diffus im Gewebe verbreiten [7]. Als probate Methode sei hier der sog. RA(„radiological alteration“)-Index zu erwähnen. Dieser stellt ein valides Mittel zur Bewertung der Gasverteilung im Körper aufgrund physiologischer Leichenveränderungen dar und erlaubt somit eine Differenzierung zwischen vitalen und postmortalen Gaseinlagerungen, etwa durch Fäulnis [10]. Fäulnisgase können die Erkennung von Stichverletzungen insbesondere in parenchymatösen Organen in der nativen PMCT deutlich erschweren, vor allem wenn andere, indirekte Zeichen wie etwa Blutansammlungen fehlen [13].

### Knöcherne Verletzungen

**Abb. 7:**
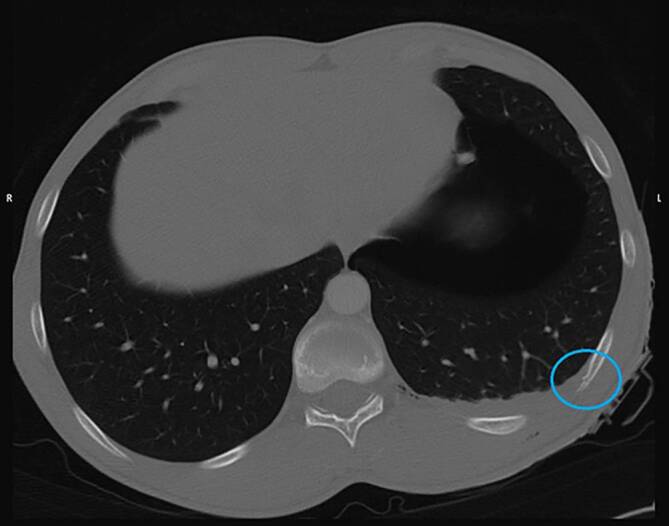
CT des Brustkorbs im Knochenfenster nach Messerstichverletzung links thorakal mit Fraktur der Rippe durch das Messer (*Kreis*)

Verletzungen der Knochen sind in der Regel radiologisch gut darstellbar und spielen in der rechtlichen Würdigung ebenfalls eine erhebliche Rolle, da sie einerseits Rückschlüsse auf ein verwendetes Tatwerkzeug zulassen [13, 16], zum anderen – insbesondere beim Durchstechen von Knochen wie dem Schädel oder dem Brustbein – auf eine erhebliche Wucht bei der Tatausführung geschlossen werden kann. Anhand derartiger Verletzungen kann der Wundwinkel bzw. Stichkanal sehr genau bestimmt werden, da eine Drehung des Tatwerkzeugs in der Verletzung aufgrund des knöchernen Widerstands kaum möglich ist [2]. Kleinere Kerben können autoptisch leicht übersehen werden bzw. Folge unvorsichtiger Obduktionstechnik sein. Insofern sollten derartige radiologischen Befunde idealerweise bereits vorab mit den Obduzent*innen besprochen werden. Knochenverletzungen durch scharfe Gewalt zeigen im Übrigen erfahrungsgemäß fremdbeigebrachte Verletzungen an, da eine sehr hohe Intensität erforderlich ist. Diese wird im Rahmen von zumeist zögerlich ausgeführten, selbst beigebrachten Stichen selten aufgebracht (Abb. 7).

## Fallstricke

**Abb. 8:**
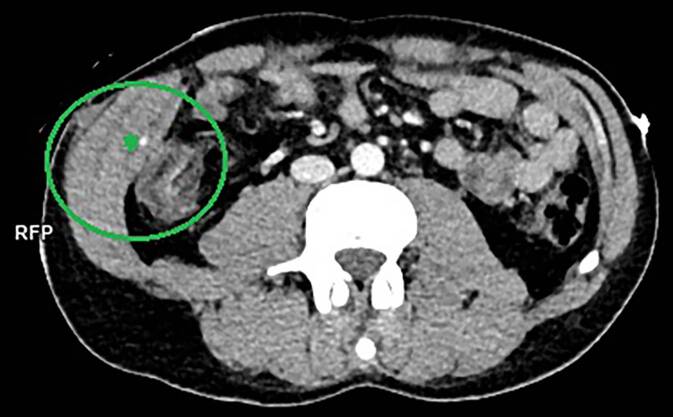
CT-Angiographie (PMCTA). Stichverletzung rechter Unterbauch mit Einblutung in das Weichgewebe, aktiver Muskelblutung (*Stern*) und Eröffnung der Bauchhöhle. Einblutungen in Peritoneum und Darm

Die radiologische Begutachtung sollte stets durch forensisch erfahrene Radiolog*innen erfolgen, idealerweise in Zusammenarbeit mit Rechtsmediziner*innen, da typischerweise Stichverletzungen eine Vielzahl an Artefakten aufweisen und zu Fehlinterpretationen führen können. Insbesondere kann sich die Beurteilung eines Stichkanals als sehr komplex erweisen, da es durch Positionsveränderungen (Stich im Stehen, CT im Liegen) zu Verschiebungen kommen kann, die den Verlauf des Stichkanals verfälschen können. Zudem kann es bei mehreren und/oder sich kreuzenden Verläufen zu Fehlern in der Einschätzung der Länge oder der Anzahl der Stiche kommen. Des Weiteren müssen Lufteinschlüsse im Herzen nicht immer zwingend Folge einer Herzstichverletzung sein, sondern können auch auf eine Luftembolie hindeuten. Falls den radiologischen Aufnahmen eine medizinische Versorgung vorausgegangen ist, muss die Befunderhebung zwingend in Zusammenschau mit sonstigen Behandlungsunterlagen erfolgen, um z. B. eine Verwechslung einer Stichverletzung mit einer Hautdurchtrennung nach erfolgter Thoraxdrainage zu vermeiden. Es ist ferner auf die Betrachtung bei üblichen Fensterungen (etwa Weichteil- und Knochenfenster) sowie die Verwendung des richtigen Faltungskerns zu achten, um auch diskrete Befunde feststellen zu können ([13]; Abb. 8).

## Fazit für die Praxis

- Derzeit stellt die Multi-Slice-Computertomographie (MSCT) zur Beurteilung von scharfer Gewalt insbesondere von Stichverletzungen die perfekte Ergänzung zur klassischen Obduktion dar. Je nach Fragestellung ist sie dieser auch als überlegen anzusehen.
- Insofern sollte – je nach Verfügbarkeit – die Durchführung einer postmortalen Computertomographie (PMCT) vor derartigen Obduktionen als obligat angesehen werden.
- Da aber neben klassischen klinischen Fragestellungen auch Fragen nach dem Tatwerkzeug, dem Stichkanal oder der Wucht beantwortet werden müssen, ist eine solche Beurteilung durch forensisch versierte Radiolog*innen, am besten gemeinsam mit erfahrenen Rechtsmediziner*innen durchzuführen.
- Ergänzend können im Strafverfahren 3‑D-Rekonstruktionen zum besseren Verständnis und zur Veranschaulichung dienen.
- Insofern sind radiologische Verfahren bei scharfer Gewalt nicht nur eine erhebliche Ergänzung und Hilfe in der täglichen rechtsmedizinischen Routine, sondern stellen mittlerweile auch ein wichtiges Instrument zur Herstellung der Rechtssicherheit dar.
